# Application of Alkyl Polyglucosides as Components of the Extraction Medium in the Preparation of a Shampoo Cosmetic Formulation Containing Substances Isolated from Red Grape Pomace

**DOI:** 10.3390/molecules30183817

**Published:** 2025-09-19

**Authors:** Tomasz Wasilewski, Zofia Hordyjewicz-Baran, Ewa Sabura, Katarzyna Malorna, Ewa Dresler, Maciej Zegarski, Natalia Stanek-Wandzel

**Affiliations:** 1 Łukasiewicz Research Network-Institute of Heavy Organic Synthesis “Blachownia”, Energetykow 9, 47-225 Kedzierzyn-Kozle, Poland; 2 Department of Cosmetology, Faculty of Medical and Health Sciences, University of Radom, Chrobrego 27, 26-600 Radom, Poland; 3 Onlybio.Life S.A., 85-825 Bydgoszcz, Poland

**Keywords:** red grape pomace, surfactants, micellar extraction, shampoo

## Abstract

This study highlights the use of alkyl polyglucosides (APGs) as sustainable and mild surfactants in cosmetic preparations, such as shampoos, following the principles of green chemistry and environmentally friendly development. APGs are non-ionic surfactants of plant origin. Their favorable dermatological and toxicological profile, as well as their high skin compatibility, make them an excellent alternative to conventional surfactants used in cosmetic products. To increase the sustainability and functionality of cosmetic preparations, the concept of loan extraction was applied, in which the extraction medium is borrowed from the final cosmetic formulation. After the extraction process, the medium enriched with the extracted compounds is returned to the cosmetic. The APGs, as part of cosmetic formulations, were used in the micellar extraction process of grape pomace, a by-product of wine production. The study evaluated the effect of different types of APGs—coco-glucoside and decyl glucoside—and their concentrations on extraction efficiency, measured by LC-MS/MS based on the content of phenolic compounds and amino acids, as well as the total phenolic content, total anthocyanin content and antioxidant activity assessed by UV-Vis spectroscopy. The designed extraction medium was then used to develop a shampoo, which showed a significantly lower zein value compared to the reference preparation without extract, indicating a reduced skin irritation potential. These results highlight the potential of APG in the development of milder, sustainable cosmetic products with the ability to extract bioactive components, supporting their use in the production of environmentally friendly cosmetics.

## 1. Introduction

Hair is an important element of physical attractiveness, and its appearance is widely recognized as an indicator of health [1]. Numerous scientific achievements in the field of hair science and hair care technology have been reported in the literature, including innovative strategies and solutions for cosmetic treatments and the development of new products [2,3,4,5,6,7,8]. Hair and scalp care are mainly based on the use of shampoos that provide effective and gentle cleansing. In recent years, shampoo has been seen not only as a purifying agent, but also as a tool to support the health and aesthetics of hair, giving it shine and making it easier to comb. In response to growing consumer expectations for multi-purpose products, and in the context of the latest marketing trends focused on the ‘natural world’, intensive research is being conducted on natural ingredients and innovative shampoo formulation techniques.

Since ancient times, people have used herbs and plant extracts to cleanse, beautify and care for their hair. Nowadays, in line with current trends, there is a growing interest in cosmetics based on natural ingredients [9,10,11]. The interest in these products is justified by their relatively low price and limited risk of side effects, as confirmed by toxicological studies [12,13].

Many plants offer beneficial effects on hair due to their content of vitamins, amino acids, sugars, phytohormones, bioflavonoids, fruit acids, and essential oils, making them valuable ingredients in natural shampoos. Consequently, numerous studies are being conducted on the extraction process and formulation of shampoos based on natural raw materials. Agricultural waste is particularly important as a cost-effective and eco-friendly alternatives to traditional plant extracts. For example, grape pomace from the wine industry is especially interesting. Winery wastes are rich in bioactive compounds and amino acids, and can be used to produce value-added components for cosmetic applications [14,15,16,17,18,19]. For this purpose various extraction processes are developed [20,21,22,23,24,25,26]. It is essential to ensure that such products are safe, effective for long-term use, and provide a mild cleansing action and an aesthetic appearance. Developing cosmetic formulations from natural ingredients is challenging, mainly due to the importance of selecting the appropriate raw materials and developing innovative formulation techniques [20]. The use of alkyl polyglucosides (APGs)—non-ionic surfactants derived from renewable raw materials such as sugars and vegetable oils—is particularly interesting in this context. The structure of APGs contributes to their excellent surface activity and micelle formation in aqueous solutions. These micelles are particularly effective in solubilizing hydrophobic bioactive compounds from plant matrices [27]. The amphiphilic properties of APGs enable solutions of these compounds to achieve what often requires the use of mixed solvent systems, providing a simplified but effective extraction mechanism. The amphiphilic structure increases their usefulness in the extraction of compounds with varying degrees of polarity, which is crucial in the case of complex plant-derived materials. The ability of APGs to aggregate into micelles is a key property for the selective extraction of a wide range of bioactive substances. The molecular structure of APGs, including differences in alkyl chain length and degree of polymerization, has a direct impact on their performance as surfactants. Different APGs exhibit varying degrees of solubilization efficiency for phenolic compounds, anthocyanins, and other functional bioactive substances. The ability to precisely tailor the properties of surfactants to specific compounds makes APGs very advantageous in applications requiring customized extraction processes [28,29,30,31]. The selectivity of APG-based extraction processes can be fine-tuned by manipulating several key parameters. The alkyl chain length of APGs influences micelle size and hydrophobicity, which in turn affects their affinity for compounds of different polarities. Additionally, factors such as APG concentration, pH and ionic strength can alter micellar structure and solubilization capacity. While the interfacial behavior of alkyl polyglycosides is relatively temperature-independent [32], temperature may still indirectly influence extraction performance by affecting matrix solubility and diffusion processes. This tunability is particularly important when working with complex plant matrices containing both hydrophilic and hydrophobic bioactives. In addition, APGs provide an emulsion stabilizing effect in the initial stages of extraction, which is essential for maintaining the integrity of bioactive compounds and further increases the stability of the final product. Furthermore, compared to traditional ionic surfactants, the non-ionic nature of APGs reduces the risk of irritation or destabilization of sensitive bioactive molecules. This mildness is particularly beneficial to their use in cosmetic preparations intended to be applied to the skin, ensuring that the functional properties of the extracted ingredients are preserved while minimizing undesirable interactions.

The environmental profile of APGs further emphasizes their suitability for use in cosmetics. They are derived from renewable plant sources, are biodegradable and have low toxicity, which is in line with the principles of sustainable development. As emphasized by Moldes et al. [31], compliance with green chemistry principles not only reduces environmental impact but also meets regulatory and consumer requirements for eco-friendly, safer cosmetic ingredients. The production of APGs requires the use of plant-based fatty alcohols and glucose, reflecting a circular economy approach that utilizes renewable resources and reduces dependence on fossil-based raw materials. Their high biodegradability also minimizes persistence in the environment and reduces toxicity to the aquatic ecosystem, addressing key concerns associated with the use of cosmetics. The mildness and low toxicity of APGs offer significant dermatological benefits. Compared to conventional, also known as aggressive, surfactants such as sodium lauryl sulphate, APGs reduce the potential for skin irritation, thereby increasing the suitability of cosmetic products for people with sensitive skin. The use of APGs in extraction processes therefore complies with international standards for ecological extraction and product safety. Furthermore, APG’s ability to meet eco-labeling requirements ensures its suitability for use in cosmetic formulations intended for environmentally conscious markets.

In our previous study, we demonstrated that extraction using decyl glucoside enables the effective leaching of cosmetically valuable compounds from red grape pomace. The extract obtained was used to develop shower gels following the idea of borrowing the compound from the final cosmetic formulation to perform the extraction process (loan extraction). The addition of the extract to cosmetic preparations had a positive effect on the functional and dermatological properties of these products [33]. Following the loan extraction concept, this study investigated the effect of the concentration and type of alkyl polyglucosides on the efficiency and selectivity of the extraction of bioactive compounds from red grape pomace for use in cosmetic preparations. Increasing the content of bioactive compounds can be achieved by raising the extract content in the final product, but also during the preparation of the extract itself by designing more concentrated systems. In this study a high concentration of plant material was applied for extraction process, Additionally, in the present work, the loan extraction was specifically prepared for use in shampoo formulations. Although both products, shower gel and shampoo, are used for washing, they contain different ingredients and functions, tailored to different needs of the scalp and body. Therefore, developing different kinds of cosmetics products is very important.

Based on the high concentrations of extracts obtained, a shampoo formulation was developed and the benefits of adding the extracts to its properties were demonstrated.

## 2. Result and Discussion

### 2.1. Evaluation of Extraction Efficiency and Bioactivity of Grape Pomace Extracts Using Alkyl Polyglucosides

#### 2.1.1. Extraction Process

The primary objective of the study was to develop cosmetics enriched with bioactive compounds derived from plant extracts with particular attention to safety for humans and the environment. The proposed method of preparing cosmetics includes an idea of ‘loan extraction’ (LE), where components borrowed from the final product are used. With this pragmatic approach, no additional purification step of the extract is needed, resulting in fewer chemicals being released into the environment and thus improving human and environmental safety [33].

As part of our research, we have developed a shampoo formulation based on natural, commercially available ingredients selected in line with current consumer trends for natural products. The product contains non-ionic surfactants, which were initially used in micellar extraction to extract bioactive compounds from plant material.

A micelle-based extraction agent containing only ingredients intended for use in the final product was developed for this purpose. It was intended to have a dual function: both as a cleansing agent in final hygiene cosmetics and as an effective extraction agent. Surfactants designed for use in the final composition of the cosmetic product—shampoos—were selected for the extraction process. Due to their amphiphilic properties, surfactants form association aggregates (micelles) in aqueous solutions. Inside these aggregates, hydrophobic substances can be solubilized [33].

It is known that bioactive substances present in grape pomace, which are valuable from a cosmetic point of view, are a mixture of compounds with both hydrophilic and hydrophobic structures. For the extraction process, APG-based surfactants were used. In aqueous solution, APG can form association aggregates (micelles), which allow the solubilization of hydrophobic substances. This phenomenon plays a key role in both the washing process and micellar extraction [34,35,36,37,38,39]. Extraction efficiency tests were carried out for different concentrations of coco-glucoside solution, namely 1%, 2%, and 4% (*w*/*w*). The results obtained were compared with the efficiency achieved using a 2% (*w*/*w*) solution of decyl glucosides.

**Figure 1:**
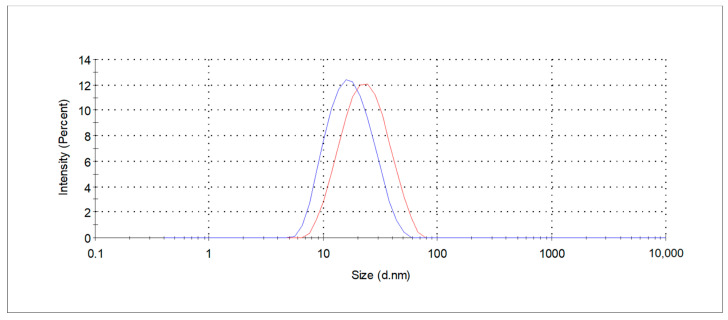
Size distribution curves of the extraction medium, determined by DLS measurements (blue line—DG; red line—CG).

Prior to extraction process the micellar extraction medium were characterized in terms of the size of the aggregates formed in the solution and their distribution, by DLS measurements. The tested concentration of 1% (*w*/*w*) revealed the formation of aggregates, indicating that the aggregation process has begun and that the concentration exceeds the CMC values of the specific surfactant. The obtained size distribution measured as hydrodynamic diameter is shown in Figure 1. A monomodal size distribution profile was found for the tested surfactants solution with the z-average hydrodynamic diameter of 16 nm (PDI 0, 16) and 21 nm (PDI 0, 16) for decyl and coco-glucoside, respectively. The presence of nanometer-sized entities indicates the formation of small micelles, whereas coco-glucoside is characterized by larger micellar structures. The data obtained for other concentrations are presented in Table 1. In a 4% (*w*/*w*) aqueous solution of decyl glucoside, a bimodal particle size distribution was observed, indicating the formation of micelles and, most likely, clusters of micelles.

Nanometer-sized particles provide an advanced platform for the effective leaching or stabilization of various active ingredients. Their small size enables precise transport to specific action sites, while minimizing the risk of disrupting their biological activity. They are especially useful for delivering poorly soluble or short-lived natural compounds.

The use of APG solution in extraction processes facilitates obtaining an extraction medium containing nanometric aggregates (micelles), which in the bulk phase ensure effective leaching of valuable bioactive compounds from plant material. Since the extraction medium is a ready-to-use component in the final cosmetic product, the extraction process has been defined as ‘loan extraction’, meaning the use of substances borrowed from the final product to carry out the extraction.

**Figure 2:**
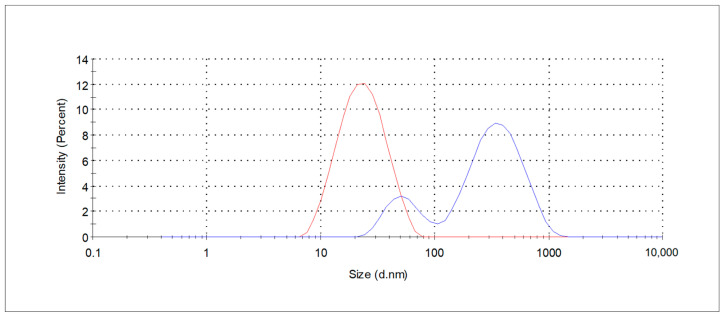
Size distribution curves of the CG_1p extraction medium (red line) and respective GPE_CG_1p (blue line), determined by DLS measurements.

The effects of concentration and type of APGs on extraction efficiency were evaluated by extract analyzing regarding bioactive compounds and antioxidants properties using LC-MS/MS and UV-Vis spectroscopy. The resulting extracts, labeled as GPE_CG_1p, GPE_CG_2p, and GPE_10pCG_4 for 1%, 2%, and 4% medium solutions of coco-glucoside, respectively, as well as GPE_DG_2p for a 2% decyl glucoside medium solution, were used for further studies on their composition and potential application in cosmetic products, such as shampoos. The size distribution measured as hydrodynamic diameter for 1% coco-glucoside solution before and after extraction is shown in Figure 2. After the extraction process the change in size distribution profile is observed indicating formation of different form of association aggregates. Increasing hydrodynamic diameters of aggregates (57 nm (20%) and 389 nm (80%), PDI 0.59) and bimodal size distribution resulting from the bioactive compounds extraction into surfactants’ micelles were noticed.

Bioactive compounds with aromatic rings exist in solution in an anionic state with one or two charges. APGs, on the other hand, are nonionic surfactants, so electrostatic interactions between them can be neglected. The water solubility of bioactive compounds is low, so the extraction into APG micelles will most likely occur via hydrophobic interactions. With the formation of APGs micelles, the solubilized space and capacity of APGs micelle both increase, hydrophobic force becomes the main driving force for the flavonoids extraction in the micelles. The ability to extract and solubilize insoluble or poorly soluble compounds is an inherent property of surfactants. Strictly hydrophobic substances are incorporated into the micellar core, while partially water-soluble substances with low to moderate hydrophobicity can also solubilize in the micellar corona between the hydrophilic chains [40,41,42]. The resulting dispersion is characterized by the presence of nanometric micelles.

The solubilization capacity of a surfactant system is directly linked to its ability to form micelles, which is triggered by reaching the CMC value. Therefore, increasing the surfactant concentration above the CMC is the primary way to enhance the solubilization of hydrophobic substances. However, there is an optimal range of surfactant concentration. Excessively high concentrations can lead to undesirable effects, such as decreased stability, increased viscosity, and other negative properties.

It is currently known that the particle sizing approach is limited and does not offer satisfactory predictability or practical applicability with respect to optimal surface tension reduction and phase behavior. However, the confirmation of the presence of aggregates and their size characterization is still the starting point for considering the self-assembly process and solubilization capability.

#### 2.1.2. Determination of Selected Compounds by UPLC-MS/MS

The content of bioactive compounds in extracts obtained from grape pomace—prepared using micellar extraction for GPE_CG 1p, GPE_CG 2p, GPE_CG 4p, and GPE_CG 2p—is summarized in Table 2.

##### Determination of Phenolic Compounds

Analysis of the results obtained allowed evident correlation to be observed in terms of the composition of the extracts. Polyphenols, i.e., catechin and epicatechin, were the dominant compounds in all samples—irrespective of the medium concentration used. The medium containing 1% coco-glucoside (GPE_CG 1p) was the most effective in solubilizing these compounds, with catechin and epicatechin concentrations of 946 mg/L and 1805 mg/L, respectively. A similar correlation was observed for gallic acid, where it assumed a concentration of 7.86 mg/L. The concentrations achieved here markedly surpass those reported by A. Chiavalori et al. [43] as well as those obtained via other extraction methods [44], suggesting that the surfactant-based extraction described in this study could be exceptionally effective.

GPE_CG 4p was the most effective in extracting galloylated derivatives such as catechin gallate (5.92 mg/L) and epicatechin gallate (14.5 mg/L). These results support earlier observations that higher surfactant concentrations can facilitate the extraction of more hydrophobic or structurally complex phenolics [31,35]. Similar patterns were described for surfactants like Brij S20, which shares amphiphilic properties with coco-glucoside and showed increased affinity for complex polyphenols [45].

Interestingly, GPE_CG 2p, while less efficient than the 1p and 4p extracts, still outperformed GPE_DG 2p across most polyphenolic parameters. However, GPE_DG 2p exhibited a markedly higher concentration of quinic acid (2.57 mg/L) compared to 0.346–0.372 mg/L in CG-based samples, suggesting that surfactant structure significantly affects the selectivity of compound solubilization [38,46]. This reinforces the importance of tailoring surfactant choice and concentration based on the desired bioactive profile.

##### Determination of Amino Acids

Proper skin care requires the use of cosmetic formulations enriched with natural bioactive compounds, including amino acids. These compounds play a fundamental role in maintaining skin hydration, elasticity, and regeneration [47].

Among the amino acids analyzed, the most effective solubilization was observed for L-leucine, with the highest concentration observed in the GPE_CG 4p extract (42.4 mg/L). In the remaining samples, its content ranged from 29.4 to 30.1 mg/L. This amino acid is known to stimulate collagen production and support skin firmness and repair processes [41]. For selected amino acids, a correlation was also observed indicating that an increase in surfactant concentration translates into higher concentrations of a chosen amino acid in the extract. For L-tryptophan, for example, the values were GPE_CG 1p (21.8 mg/L), GPE_CG 2p (24.3 mg/L), and GPE_CG 4p (27.2 mg/L), respectively. A similar trend was observed for L-Histidine (2.05–2.30 mg/L), L-Phenylalanine (11.8–18.2 mg/L), and L-Methionine (2.51–4.61 mg/L). Analysis of the results revealed an interesting occurrence for L-Lisine, whose concentration in the GPE_CG 2p extract was 30.6 mg/L—a value significantly higher compared to the other extracts with different surfactant concentrations. A different kind of the correlation was found for L-Valine, L-Leucine, and L-Aspartic acid. For these, the lowest concentrations were found in GPE_CG 2p extracts, while the highest concentrations were found in GPE_CG 4p, which may indicate a more complex extraction mechanism for these amino acids.

The study results for L-leucine showed its highest concentration in the GPE_CG 4p extract (42.4 mg/L). In the remaining samples, its content ranged from 29.4 to 30.1 mg/L. This amino acid is known to stimulate collagen production and support skin firmness and repair processes [48]. For selected amino acids, a correlation was also observed indicating that an increase in surfactant concentration translates into higher concentrations of a chosen amino acid in the extract. For L-tryptophan, for example, the values were GPE_CG 1p (21.8 mg/L), GPE_CG 2p (24.3 mg/L), and GPE_CG 4p (27.2 mg/L), respectively. A similar trend was observed for L-Histidine (2.05–2.30 mg/L), L-Phenylalanine (11.8–18.2 mg/L), and L-Methionine (2.51–4.61 mg/L). Analysis of the results revealed an interesting occurrence for L-Lysine, whose concentration in the GPE_CG 2p extract was 30.6 mg/L—a value significantly higher compared to the other extracts with different surfactant concentrations. A different kind of the correlation was found for L-Valine, L-Leucine, and L-Aspartic acid. For these, the lowest concentrations were found in GPE_CG 2p extracts, while the highest concentrations were found in GPE_CG 4p, which may indicate a more complex extraction mechanism for these amino acids.

Finally, a comparison between CG and DG extracts revealed that CG-based systems consistently resulted in significantly higher amino acid yields across all tested compounds. This aligns with earlier findings that highlight the superior micelle-forming capacity of coco-glucoside and its favorable interaction with complex plant matrices [31,49].

This study successfully quantified the amino acid content available in the laboratory samples and elucidated the significance of amino acids in hair cosmetic products. However, the analysis did not include several amino acids that have well-established benefits for hair structure and hydration, such as cysteine, arginine, serine, proline, and glycine. Including these key amino acids in future research would enable a more comprehensive elucidation of their roles in shampoo formulations and their overall impact on hair care efficacy. Such an expanded analysis would undoubtedly be of scientific and practical interest for the future development of hair hygiene and care products.

##### Determination of Anthocyanins

Empirical evidence from our previous micellar extraction studies [50] employing decyl glucoside further illustrates the importance of optimizing the type and concentration of surfactants when targeting anthocyanin recovery. The anthocyanin content in extracts obtained from grape pomace is presented in Table 3 and reflects both the diversity and relative abundance of individual compounds under different concentrations and type of the surfactants.

The UPLC-ESI-MS/MS analysis in positive-ion mode facilitated the identification of nine anthocyanin compounds in grape pomace extracts. The predominant forms were 3-O-glucosides and their acylated derivatives, indicating a characteristic anthocyanin profile of grape-derived matrices. A comparative evaluation of relative peak intensities revealed a general increase in anthocyanin content with higher concentrations of the surfactant in extraction medium. Notably, coumaroylated anthocyanins—such as malvidin 3-(6″-coumaroyl)-glucoside and peonidin 3-(6″-coumaroyl)-glucoside—showed the highest fold changes with increasing surfactants concentration, suggesting that these more lipophilic derivatives are particularly responsive to extraction parameters.

Furthermore, a comparison of GPE_CG_2p and GPE_DG_2p extracts showed a higher relative content of anthocyanin compounds in GPE_CG_2p. Overall, the results emphasize that both the concentration of the extract and the nature of the extract (GPE_CG vs. GPE_DG) have a significant impact on the qualitative and quantitative composition of anthocyanins. These findings are important for the valorization of grape pomace as a source of bioactive compounds and for the optimization of extraction process for the targeted enrichment of anthocyanin compounds.

#### 2.1.3. Total Phenolic Content (TPC), Total Anthocyanin Content (TAC), and Antioxidant Capacity (DPPH, ABTS)

One of the aims of this study was to assess the influence of CG concentration in the extraction medium (Table 4) on the recovery of phenolic compounds and antioxidant activity of red grape pomace extracts, and to compare these results with those obtained using DG—Table 5. Total phenolic content (TPC), total anthocyanin content (TAC), and antioxidant capacity (DPPH and ABTS assays) were measured for each extract. The results, expressed as mean values ± standard deviation (SD), revealed notable differences depending on both the surfactant concentration and type, providing valuable insight into the effectiveness of alkyl polyglucosides in aqueous micellar extraction systems for potential use in cosmetic formulations.

The results of this study demonstrate that the concentration of CG markedly influences the efficiency of extracting bioactive compounds from red grape pomace, with 2% CG (GPE_CG 2p) yielding the most favorable balance of extract composition and functional properties. At this concentration, the extract showed the highest total anthocyanin content (226.8 ± 5.3 mg Cyd-3-glu/L) and the strongest antioxidant activity, measured by both DPPH (3598.6 ± 14.2 mg TE/L) and ABTS (3708.6 ± 49.6 mg TE/L), alongside a high total phenolic content (TPC: 2747.7 ± 37.4 mg GAE/L). These findings are in line with earlier studies showing that moderate APG concentrations enable optimal micelle formation and solubilization of a wide range of phenolic antioxidants, including anthocyanins [27,31,35].

For GPE_CG_4p, despite a slight increase in TPC (2780.1 ± 31.2 mg GAE/L), antioxidant activity declined (DPPH: 3152.5 ± 50.1 mg TE/L; ABTS: 3411.7 ± 42.3 mg TE/L), and anthocyanin content dropped to 203.9 ± 5.1 mg Cyd-3-glu/L. This suggests that phenolic enrichment at higher CG concentrations may not directly translate to greater functional efficacy. As shown by Moldes et al. [31] and Bogdanovic Markovic et al. [27], excess surfactant may alter micelle structure or increase solution viscosity, negatively affecting compound accessibility and extraction kinetics. Moreover, high CG levels may promote the extraction of more polymerized or less reactive phenolics with lower antioxidant potential, which is reflected in the reduced scavenging capacity observed at a CG concentration of 4%. These observations are consistent with the findings of prior studies using surfactant-based systems, where micelle over-saturation and internal structural rearrangements above the CMC reduce the availability of solubilizing domains for bioactives [28,30,31]. Parande et al. [38] further emphasize that excessive APG concentrations can impair extraction through increased medium viscosity and reduced diffusion rates.

Importantly, antioxidant capacity was evaluated using two complementary assays—DPPH and ABTS—which assess the radical scavenging potential of extracts through different mechanisms. As previously reported by Brand-Williams et al. [51] and Re et al. [52], these methods are widely accepted for profiling the antioxidant power of phenolic-rich extracts, with ABTS being more broadly reactive with both hydrophilic and lipophilic antioxidants. The strong agreement between both assays in this study further supports the robustness of the results and confirms the functional superiority of the 2% CG extract.

From a practical standpoint, extraction with 4% CG introduced significant operational issues. The high concentration of surfactants in extraction medium may exhibit intense foaming, which hindered mixing, filtration, and handling of the plant material—an observation similarly reported by Wojcieszak et al. [36] in APG-rich micellar systems. This poses a barrier to process scalability and underlines the need to optimize not only chemical efficacy but also technical feasibility when designing sustainable cosmetic extraction protocols.

**Table 5:** Comparison of surfactant type (decyl glucoside vs. coco-glucoside) on total phenolic content (TPC), total anthocyanin content (TAC), and antioxidant activity (DPPH, ABTS) of red grape pomace extracts (mean ± SD).

Extract	Surfactant Type	TPC [mg GAE/L]	TAC [mg Cyd-3-glu/L]	DPPH [mg TE/L]	ABTS [mg TE/L]
GPE_DG 2p	DG	1476.5 ± 19.8	160.0 ± 7.2	2097.3 ± 48.9	1820.7 ± 22.1
GPE_CG 2p	CG	2261.6 ± 25.9	208.4 ± 5.9	2898.8 ± 50.3	2972.1 ± 25.1

Based on the data from Table 5, it was observed that the type of surfactant applied has a significant impact on the efficiency of bioactive compound extraction from red grape pomace. The data clearly show that CG resulted in higher yields of both total phenolic content (TPC: 2261.6 ± 25.9 mg GAE/L) and total anthocyanin content (TAC: 208.4 ± 5.9 mg Cyd-3-glu/L), compared to DG (TPC: 1476.5 ± 19.8 mg GAE/L; TAC: 160.0 ± 7.2 mg Cyd-3-glu/L). Antioxidant activity, evaluated using DPPH and ABTS assays, was also significantly higher in extracts obtained with CG (DPPH: 2898.8 ± 50.3 mg TE/L; ABTS: 2972.1 ± 25.1 mg TE/L) than with DG (DPPH: 2097.3 ± 48.9 mg TE/L; ABTS: 1820.7 ± 22.1 mg TE/L).

These differences can be attributed to the distinct molecular characteristics of the surfactants. CG typically contains longer alkyl chains (C12–C14) compared to DG (C8–C10), which enhances micelle size and stability, thus improving the solubilization of a broader range of phenolic compounds, including moderately hydrophobic and amphiphilic molecules [27,31,35]. According to Bogdanovic Markovic et al. [27] and Moldes et al. [31], this structural advantage allows CG to more efficiently penetrate plant matrices and form stable colloidal systems that retain bioactive compounds during extraction. Moreover, as noted by Wojcieszak et al. [36], CG exhibits a stronger ability to stabilize emulsions and maintain dispersion homogeneity, further contributing to its superior performance.

In contrast, DG forms smaller micelles with lower capacity for encapsulating complex phenolic structures. Parande et al. [38] and other researchers [28,31] emphasized that such differences in micelle morphology, hydrophilic–lipophilic balance (HLB), and interfacial behavior directly influence extraction yields. Additionally, the greater antioxidant activity observed with CG extracts may reflect not only the higher concentration of phenolics but also a more favorable composition of active compounds with stronger radical scavenging properties, as also suggested by the comparative performance in both DPPH and ABTS assays [51,52].

In conclusion, the use of 2% coco-glucoside extraction medium provided the most effective balance between extraction efficiency, antioxidant activity, and process feasibility. Compared to decyl glucoside, coco-glucoside enabled higher recovery of phenolics and anthocyanins, confirming its superior performance in micellar extraction of red grape pomace. These findings underscore the importance of optimizing both the type and concentration of biodegradable, non-ionic surfactants in the development of green and scalable extraction methods for sustainable cosmetic applications [27,28,31,32,33,34,35,36,37,38].

### 2.2. Design of Model Cosmetics

The aim of the study was to evaluate the effect of the extract additive on the properties of the final cosmetic products. For this purpose, shampoo formulations containing no extract (labeled Sh_E_0p) and with a 10% addition of red grape pomace extract (labeled Sh_E_10p) were developed. The added extract was prepared using a loan extraction method involving coco-glucoside, borrowed from a cosmetic formulation for micellar extraction. This approach aimed to obtain a product that meets safety and efficacy standards. The detailed composition of the developed preparations is presented in Table 6.

The shampoo formulation contains a mixture of substances, including primary and secondary surfactants for cleaning, thickening agents, solvents, conditioning agents, pH regulators, and other ingredients such as fragrances and dyes to enhance the attractiveness of the product [2].

Common cleansing ingredients are anionic surfactants, which are characterized by a negative charge on the hydrophilic polar group. They are highly effective at removing sebum and impurities, but due to their strong cleaning properties, they can increase negative electrical charges on the hair surface, causing frizz and friction. In order to reduce potential damage and achieve a milder cleansing effect, secondary surfactants, such as non-ionic and amphoteric ones, are usually added to the preparations [53]. Non-ionic surfactants do not have an electrical charge in aqueous solutions, are less aggressive than anionic surfactants and, thanks to their eudermic properties, are widely used as emulsifiers and solubilizes in cosmetics.

In recent years, there has been a trend towards the use of plant-derived raw materials such as glucosides, citrates, sulfosuccinates and protein hydrolysates. In our research, we examines the use of non-ionic surfactants from the APG group, where CG was selected based on the best results of the extraction efficiency assessment. Scientific research results emphasized the effectiveness of CG in improving extraction efficiency and, as reported in the literature, in preparation stability [35]. As noted by Wojcieszak et al. [36], CG enables the formation of colloidal dispersions with droplet sizes below 1000 nm, which indicates high emulsion stability and effective delivery of compounds. This property is related to negative zeta potential values (up to −38.67 mV), which prevent droplet aggregation and maintain stability in the extract matrix. Additionally, low polydispersity indices ensure uniformity in droplet size and distribution of bioactive substances, which translates into improved sensory properties and consistent product performance. The physicochemical advantages of CG offer practical benefits that are invaluable in cosmetic applications. Controlled release of active compounds and compatibility with a wide range of cosmetic ingredients highlight its versatility. These features not only facilitate extraction but also expand the scope for innovation in advanced formulations, such as emulsions containing complex plant-based matrices. Furthermore, APG’s dual role as extraction agents and stabilizers fill the gap between functional ingredient recovery and product development, enabling seamless integration into cosmetic formulations [37].

Amphoteric surfactants have the ability to control their charge depending on the pH of the solution, which makes them extremely mild and dermatologically compatible. They have good foaming, detergent, and wetting properties and are an important ingredient in reducing the aggressiveness of final products based on anionic surfactants. Commonly used compounds in this group are alkyl amidopropyl betaines and alkyl betaines [53].

Cationic surfactants are widely used as hair conditioners because they can neutralize negative hair charges after washing, resulting in reduced frizz and improved hair condition. Due to the low isoelectric point of hair (pH 2.15–3.17), they show softening and often bacteriostatic properties [54].

Shampoo formulations also include substances that ensure the proper consistency and stability of the product and improve its performance. Due to the high water content, it is necessary to add preservatives to prevent the growth of microorganisms. Other additives include thickeners such as electrolytes, cellulose derivatives, and natural gums, which increase the viscosity of the product [55]. pH regulators adjust the pH of the shampoo to the natural pH of the scalp and hair (5.5). Sequestrants protect the ingredients from unwanted reactions. In addition, fragrances and colorants are used to improve the sensory properties [56]. In our case, the color of the prepared shampoo is naturally derived from anthocyanins extracted from red grape pomace.

Other important ingredients are functional components that give the shampoo properties tailored to the specific needs of different hair types, taking into account their physiological characteristics.

The important part of this study was to use the concept of loan extraction. The proposed method for preparing cosmetics based on plant extracts is grounded in an extraction process involving “extraction using components borrowed from the final product”. This pragmatic approach eliminates the need for additional purification of the obtained extract from auxiliary substances, thereby contributing to the reduction in chemical substances introduced into the environment and enhancing safety for both humans and the environment. As the extraction medium, a solution of surfactant compounds was utilized. Due to their amphiphilic properties, these surfactants form associative aggregates (micelles) in aqueous solutions. Within these aggregates, it is possible to solubilize hydrophobic bioactive compounds extracted from plant material.

Incorporating red grape pomace extract into a cosmetic formulation such as shampoo offers several scientifically supported advantages attributable to its rich phytochemical composition. As indicated in performed studies, such extracts are abundant in polyphenolic compounds and amino acids, exhibiting antioxidant properties. The inclusion of this extract in shampoo formulations can enhance the product’s functional efficacy by providing protective effects against oxidative stress induced by environmental factors such as UV radiation and pollution, thereby promoting healthier scalp and hair. Moreover, the antioxidant activity can help mitigate damage to hair fibers, reducing brittleness and promoting strength and resilience. The extract’s natural bioactive compounds can also improve the overall hair quality by enhancing shine, smoothness, and manageability, owing to their potential to strengthen hair shafts and improve hydration levels. Compared to conventional shampoos, which primarily focus on cleansing and may contain synthetic surfactants and preservatives, shampoos formulated with red grape pomace extract tend to offer added functional benefits rooted in natural antioxidant and bioactive principles. These benefits include increased protection against oxidative damage, reduced scalp irritation, and potential long-term improvements in hair health. Additionally, such formulations appeal to consumers seeking natural and sustainable cosmetic products, aligning with current trends toward eco-friendly and health-conscious personal care.

The prepared products were evaluated for the effect of the extract addition on their basic physicochemical properties. The results obtained are summarized in Table 7.

#### 2.2.1. Stability

Cosmetic products, i.e., the developed shampoos, demonstrated mechanical and microbiological stability. After centrifugation at 5000 rpm for 30 min, they retained their homogeneity, confirming their resistance to phase separation. As part of thermal cycle resistance testing, samples were subjected to a series of temperature changes including exposure to −18 °C for 24 h, followed by thawing at room temperature, then exposure to 40 °C for 24 h, followed by a return to room temperature for another 24 h. No changes in the structure or physical properties of the product were observed during three repeated cycles.

**Figure 3:**
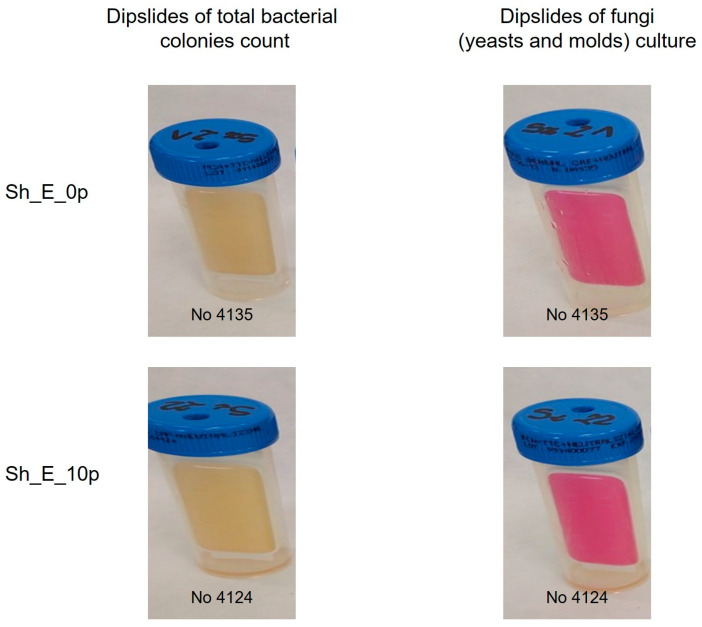
Appearance of microcount^®^ duo plates after 5 days of incubation time.

In addition, microbiological tests using the dual plate method showed that the products comply with applicable safety standards, with no growth of bacteria or fungi (molds and yeast), confirming their microbiological stability under the test conditions. In Figure 3 the resulting plates for microbiological stability testing are presented.

#### 2.2.2. Viscosity

Controlling viscosity is crucial in the cosmetics industry because it significantly affects both the production process and the final quality of the product as perceived by consumers. Viscosity management is mainly based on selecting the appropriate surfactants and viscosity modifiers that enable precise adjustment of the final product properties. Achieving and maintaining the target viscosity is not only a technical requirement but also crucial for ensuring optimal product dosing and application. In the studies conducted, no changes in the viscosity was observed after the addition of the extract (Table 7). The presence of active compounds in the extract has no effect on the thickening ability of the viscosity modifiers in the final product.

#### 2.2.3. Foaming Properties

Table 7 presents the results of foaming ability and foam stability tests for the developed shampoos. Data analysis shows that the tested products were characterized by high foaming ability and foam stability. The addition of the extract reduced the volume of foam generated, but increased foam stability. Extracted compounds can influence the aggregation behavior of surfactants in formulation. Specifically, the addition of the extract was observed to reduce foaming capacity, suggesting the formation of less stable micellar or surface structures. Flavonoids, being polyphenols with a high capacity to form stable complexes with other compounds, may interact with the hydrophilic groups of surfactants through the formation of shields or aggregates. This limits their free migration to the air-liquid interface. Such interactions decrease the surface activity of the surfactants and, consequently, reduce their ability to lower surface tension and generate stable foam. Furthermore, amino acids—especially those containing polar groups or charges—can form hydrogen bonds or electrostatic interactions with the functional groups of surfactants, affecting their dispersion and spontaneous movement toward the phase interface. As a result of these molecular interactions, the availability of surfactants at the surface is restricted, weakening their foaming properties. Conversely, more stable foam was also generated under certain conditions. These phenomena may be attributed to the polyphenolic compounds’ ability to cross-link surfactant molecules at the air–water interface, thereby enhancing the structural integrity and resistance of air bubbles against coalescence and collapse.

#### 2.2.4. Irritating Potential

As documented in scientific literature, adding natural bioactive compounds to cosmetic formulations can reduce their irritating potential and thus improve their safety for the skin [57,58,59]. In particular, the presence of phenolic compounds and amino acids in red grape pomace extracts may promote their interaction with surfactant micelles, resulting in increased micelle size and stabilization of micellar structures. These structural changes are associated with a reduction in the irritating effect of surfactants. In the formulated shampoos, an approximately 5% decrease in irritation potential was observed following the incorporation of 10% (*w*/*w*) grape pomace extract.

## 3. Materials and Methods

### 3.1. Materials

All standards used were of analytical grade (≥99% purity). Analytical standards of (+)-Catechin, (-)-Epicatechin, Rutin, (-)-Catechin 3-gallate, (-)-Epicatechin 3-gallate, quinic acid L-Phenylalanine, L-Aspartic acid, L-Valine, L-Lisine, L-Tryptophan, L-Leucine, L-Threonine, L-Methionine, and L-Histidine were purchased from Merck (Darmstadt, Germany); Gallic acid, ABTS (2,2′-azino-bis(3-ethylbenzothiazoline-6-sulfonic acid) diammonium salt, and 6-hydroxy-2,5,7,8-tetramethylchromane-2-carboxylic acid (Trolox) were purchased from POL-AURA (Zabrze, Poland); and DPPH (2,2-diphenyl-1-picrylhydrazyl) was purchased from Sigma-Aldrich (St. Louis, MO, USA).

For cosmetic preparation certified vegetable-based surfactants approved for natural product manufacturing under ECOCERT and COSMOS standards were used: decyl glucoside (DG; Plantacare 2000, BASF, Ludwigshafen, Germany), coco-glucoside (CG; Plantacare 818 UP, BASF, Germany), Sodium Lauryl Sulfate (Rosulfan L, PCC Excol, Brzeg Dolny, Poland), Ammonium Lauryl Sulfate (Rosulfan A, PCC Excol, Brzeg Dolny, Poland), Cocamidopropyl Betaine (Rokamina K30, PCC Excol, Brzeg Dolny, Poland), Benzyl Alcohol, Benzoic Acid, Dehydroacetic Acid, Tocopherol (Euxyl K903, Ashland, Wilmington, DE, USA), Guar Hydroxypropyltrimonium Chloride (Dehyquart TC, BASF, Germany), sodium chloride (Krakchemia, Krakow, Poland), and distilled water.

### 3.2. Plant Material

The pomace from a mixture of Pinot Noir and Rondo red grapes was obtained from the Cwielong-Olszewski vineyard (Opole, Poland). All grapes were harvested at full ripeness (in early September 2024) and their berries were completely black/purple.

### 3.3. Preparation of Micellar Extracts from Red Grape Pomace Used as Cosmetic Component

A mechanical stirrer (CAT, R50D; M. Ziperer GmbH, Ballrechten-Dottingen, Germany) was utilized to enhance the micellar-mediated extraction process. Concentrations of 1%, 2%, or 4% (*w*/*w*) aqueous coco-glucoside and 2% (*w*/*w*) of decyl glucoside extraction medium were prepared. To prepared solution, a preservative mixture comprising benzyl alcohol, benzoic acid, dehydroacetic acid, and tocopherol was added at a concentration of 0.5% (*w*/*w*). Then, 400 g of ground grape pomace (ground using a laboratory knife mill, Cutter Mixer R5 Plus, Robot Coupe, Vincennes, France) was added to 100 g of the prepared extraction medium. The mixture was subjected to vigorous stirring at 380 rpm at room temperature for 20 min to facilitate extraction process. Post-extraction, the mixture was pre-filtered through a raw cotton filter bag with a weight of 145 g/m*^2^* using a wine press. Then the resulting filtrate was further filtered under vacuum using a Büchi V-700 vacuum pump and Nalgene^®^ bottle-top sterile filter units fitted with 0.45 μm pore size polyethersulfone membranes (Thermo Fisher Scientific Inc., Waltham, MA, USA). The filtrate was collected for further analysis.

### 3.4. DLS Methods

The size of micelles in extraction medium was determined at 22 °C using Zetasizer Nano (Malvern Instruments Ltd., Worcestershire, UK) equipped with a laser (633 nm) set at an angle of 173°.

### 3.5. The Preparation of the Shampoo Formulation

To water maintained at a temperature of 80 °C citric acid, and surfactants were added, excluding amphoteric one. In the case of the formulation containing extract, an extract was additionally incorporated into the mixture. The components were stirred until a homogeneous mixture was obtained. Subsequently, a preservative was added, followed by mixing, and then a conditioning polymer was incorporated. The mixture was stirred until a uniform homogeneous solution was achieved. Next, amphoteric surfactant and a viscosity modifier were added, and the mixture was stirred until a consistent, homogeneous mixture was formed.

### 3.6. UPLC-ESI-MS/MS Analysis of Selected Compounds in Grape Pomace Extracts

The analyses of selected compounds were performed in independent replicates. Filtered through the 0.2 μm syringe filters extracts solutions were separated using a liquid chromatograph (Sciex ExionLC AD, AB Sciex, Concord, ON, Canada) with a reverse-phase pre-column and column (Kinetex 3.5 µm XB-C18 100 Å; 100 × 4.6 mm, Phenomenex). Separation of the compounds was carried out using gradient elution, with specific details provided in a previously publication [50].

The MS detection was performed using a triple quadrupole mass spectrometer (4500 QTRAP, AB Sciex, Concord, ON, Canada) equipped with an electrospray ionization (ESI) source operating in both positive and negative ion modes. The ion source parameters were set as follows: ion spray voltage at 4500 V for positive-ion mode and −4500 V for negative-ion mode, source temperature at 600 °C; nebulizing and drying gas pressure were set at 50 psi, and curtain gas at 35 psi.

The ANALYST 1.7.2 software program was used to perform data analysis. The multiple reaction monitoring (MRM) mode was used to quantify the bioactive components for all extracts. The peak area of the most intense MRM transition was used to construct and plot a seven-point calibration curve based on linear regression for each standard within the concentration range of 1 to 100 mg/L. Stock standard solutions were prepared by accurately weighing and dissolving 10 mg of each reference compound in 10 mL of LC-MS grade methanol to obtain a final concentration of 1000 mg/L. All dilutions were performed using LC-MS grade methanol. For analysis, 1000 μL of each sample was transferred into an amber chromatographic vial, and the extracts were injected directly without prior dilution.

### 3.7. Total Phenolic Content (TPC)

The total phenolic content (TPC) was determined spectrophotometrically using the Folin–Ciocalteu (FC) method, based on the procedure originally described by Singleton et al. [60], with minor modifications as previously reported in our earlier publication [33]. In this study, the only change was a tenfold dilution of the grape pomace extract with distilled water before performing the measurements.

TPC was expressed as mg of gallic acid equivalents (GAE) per liter of extract, with measurements performed in triplicate.

### 3.8. Total Anthocyanin Content (TAC)

The total anthocyanin content of the extracts was estimated using the pH differential method, which relies on the structural transformation of anthocyanin molecules under different pH conditions [61]. The detailed procedure was described in our previous publication [50]. In the present study, the only modification consisted of a tenfold dilution of the grape pomace extract with distilled water prior to analysis.

TAC was expressed as cyanidin-3-glucoside equivalents (Cyd-3-glu) per liter of extract (mg Cyd-3-glu/L).

### 3.9. Antioxidant Activity (DPPH Test)

The antioxidant activity of the extract was determined using a modified method based on the procedure described by Brand-Williams et al. [51], with further details provided in our previous publication [50]. In the present study, the grape pomace extract was diluted tenfold with distilled water prior to analysis.

The antiradical activity was expressed as mg Trolox equivalent (TE) per liter of extract.

### 3.10. Antioxidant Activity (ABTS Test)

The ABTS assay was performed according to the method described by Re et al. [52], with the detailed procedure previously outlined in our earlier publication [50]. A tenfold dilution of the grape pomace extract with distilled water was the only methodological change applied in the current study.

The antioxidant activity was expressed in mg Trolox equivalent (TE) per liter of extract.

### 3.11. Characterization of Cosmetic Product (Liquid Soap)

#### 3.11.1. Viscosity

The viscosity of the shampoo was determined at 20 °C using a Brookfield DV2TRV rheometer equipped with a small sample adapter and a cylindrical SC4 spindle (Brookfield, WI, USA). An amount of 8 mL of sample was used in each test. The test was performed at a speed of 10 rpm. The result is the average of three measurements.

#### 3.11.2. Foaming Properties

The foaming properties of the shampoo were evaluated in accordance with the PN-74 C-04801 standard using a Ross-Miles apparatus. A 50 mL sample of a 10% (*w*/*w*) cosmetic solution was poured into the cylinder. Subsequently, 200 mL of the solution was drawn into a dropper positioned 1 m above the liquid surface. The dropper valve was then opened to release the solution into the cylinder. Foam height was measured at intervals of 1 and 10 min to assess both the foaming ability and the stability of the foam over time.

#### 3.11.3. Determination of Irritant Potential—Zein Value

To evaluate the irritant potential of the formulated shampoo, the zein number was measured. The test solution was prepared by dissolving 2 g of zein protein in 40 g of a 10% (*w*/*w*) shampoo solution. The amount of solubilized protein was quantified using nitrogen Kjeldahl analysis, expressed as milligrams of released nitrogen per 100 mL of sample, following the method described by Wasilewski et al. [62]. All measurements were conducted in triplicate, and the final zein number value was calculated as the arithmetic mean of the three independent measurements.

#### 3.11.4. Microbiological Stability

The microbiological stability of the model shampoo was assessed using Microcount^®^ Duo testers (Schülke & Mayr, Norderstedt, Germany). Samples were collected with sterile swabs and streaked onto agar plates. The plates were incubated at 28 °C for 3 days to detect bacteria and fungi, and for 5 days to assess yeasts and molds. After incubation, microbial growth was visually examined, and colony counts were conducted using the manufacturer’s standardized template to ensure precise quantification.

### 3.12. Statistical Analysis

All UPLC-ESI-MS/MS data are presented as mean ± standard deviation (SD) based on four replicates per sample (*n* = 4). Mean values were analyzed using one-way ANOVA followed by Tukey’s HSD post hoc test for multiple comparisons. Data analyses were conducted with Statistica software version 10 (StatSoft, Tulsa, OK, USA). A correlation matrix was employed to assess significant relationships between variables. Differences were considered statistically significant at *p* < 0.05.

## 4. Conclusions

The study demonstrated the potential of APGs, in particular coco-glucoside, as environmentally friendly components of extraction media in the production of cosmetics from agricultural and food industry by-products, such as red grape pomace. In an era of growing environmental awareness and trends towards natural products, the use of sustainable raw materials and environmentally friendly extraction methods, such as micellar extraction using components from the final formulation, is becoming increasingly important. This approach facilitated the effective extraction of bioactive compounds while eliminating additional time-consuming and costly operations and the emission of volatile organic compounds. The inclusion of coco-glucoside in shampoo formulations enabled the creation of stable, mild, and consumer-friendly products that combine effectiveness with ecological values. The addition of grape pomace extracts further increased the safety of cosmetics by reducing their irritating potential. The conclusions of the study emphasized that APGs, due to their amphiphilic properties, compatibility with bioactive ingredients and compliance with the principles of sustainable development, are an important solution for the future of the cosmetics industry.

Due to their excellent efficiency, waste-free process, low costs, and environmental friendliness, the loan extraction concept seems to be one of the best available technologies to obtain bioactive-rich extracts for cosmetics application. The possibility of using a wide range of surfactants in cosmetic applications, sets new trends for future research developing the concept of loan extraction.

In addition, the use of waste to obtain bioactive compounds supports innovation in the creation of natural and ecological products.

## Figures and Tables

**Table 1:** Determination of hydrodynamic diameter and polydispersity index for extraction medium—mean values ± standard deviation (*n* = 4).

Extraction Medium	Hydrodynamic Diameter (nm)	PDI
DG 1p	16.0 ± 0.5	0.16
DG 2p	13.2 ± 0.2	0.14
DG 4p	17.4 ± 0.1 1900 ± 100	0.39
CG 1p	20.7 ± 0.3	0.16
CG 2p	17.5 ± 0.5	0.17
CG 4p	15.2 ± 0.2	0.15

**Table 2:** Quantitative determination of selected compounds in red grape pomace extracts by UPLC-ESI-MS/MS—mean values ± standard deviation (*n* = 4).

	Compound	Quantification/ Confirmation Transition	Family	GPE_CG 1p [mg/L]	GPE_CG 2p [mg/L]	GPE_CG 4p [mg/L]	GPE_DG 2p [mg/L]
1	(+)-Catechin	290.9 > 139.0 290.9 > 123.0	flavonols	946 ± 18.4	681 ± 3.50	803 ± 12.0	444 ± 9.8
2	(-)-Epicatechin	290.9 > 139.0 290.9 > 123.0	flavonols	1805 ± 35.4	1340 ± 28.3	1570 ± 56.6	613 ± 12.3
3	(-)-Catechin 3-gallate	443.0 > 123.0 443.0 > 273.0	flavonols	4.91 ± 0.60	4.58 ± 0.08	5.92 ± 0.04	1.99 ± 0.37
4	(-)-Epicatechin 3-gallate	443.0 > 123.0 443.0 > 273.0	flavonols	10.27 ± 0.75	8.63 ± 0.15	14.5 ± 0.28	3.75 ± 0.20
5	Rutin	608.9 > 299.9 608.9 > 270.9	flavonols	2.13 ± 0.36	2.03 ± 0.05	1.91 ± 0.04	0.618 ± 0.004
6	Gallic acid	168.9 > 124.8 168.9 > 78.9	phenolic acid	7.86 ± 0.03	5.67 ± 0.47	5.17 ± 0.31	0.51 ± 0.10
7	D-(-)-quinic acid	190.9 > 84.9 190.9 > 93.0	phenolic acid	0.345 ± 0.23	0.372 ± 0.16	0.367 ± 0.38	2.57 ± 0.13
	Sum of phenolic compounds			2776	2042	2401	1066
8	L-Valine	118.1 > 72.0 118.1 > 55.0	amino acid	10.2 ± 0.41	9.9 ± 0.59	14.0 ± 0.57	5.4 ± 0.44
9	L-Methionine	150.1 > 103.9 150.1 > 132.9	amino acid	2.51 ± 0.11	3.43 ± 0.13	4.61 ± 0.31	0.804 ± 0.12
10	L-Tryptophan	205.0 > 188.0 205.0 > 145.9	amino acid	21.8 ± 0.28	24.3 ± 0.57	27.2 ± 0.49	15.3 ± 0.06
11	L-Leucine	132.1 > 86.0 132.1 > 44.0	amino acid	29.8 ± 0.21	29.4 ± 0.21	42.4 ± 0.28	30.1 ± 0.58
12	L-Histidine	156.1 > 110.0 156.1 > 82.9	amino acid	2.05 ± 0.01	2.56 ± 0.04	2.30 ± 0.01	n.d. *
13	L-Threonine	120.1 > 74.0 120.1 > 56.0	amino acid	5.67 ± 0.06	4.82 ± 0.09	6.04 ± 0.10	4.51 ± 0.02
14	L-Lisine	147.1 > 84.0 147.1 > 130.0	amino acid	20.7 ± 0.14	30.6 ± 0.21	24.0 ± 0.14	22.3 ± 0.24
15	L-Phenylalanine	163.9 > 147.0 163.9 > 103.0	amino acid	11.8 ± 0.35	14.7 ± 0.00	18.2 ± 0.71	11.2 ± 0.33
16	L-Aspartic acid	131.8 > 88.0 131.8 > 114.9	amino acid	2.82 ± 0.11	2.50 ± 0.02	4.19 ± 0.29	1.18 ± 0.08
	Sum of amino acids			107.2	122.1	142.9	90.8

* n.d.—not detected.

**Table 3:** Anthocyanin compounds identified using UPLC-ESI-MS/MS in positive-ion mode in extracts.

	Compound	Molecular Formula	Molar Mass [Da]	Precursor Ion *m*/*z*	Main Product Ion MS^2^ [*m*/*z*]	GPE_CG_4p/ GPE_CG_1p	GPE_CG_4p/ GPE_CG_2p	GPE_CG_2p/ GPE_DG_2p
1	Cyanidin 3-glucoside (Cy 3-glc)	C_21_H_21_O_11_^+^	449	449 [M + H]^+^	287 [M-C_6_H_11_O_5_]^+^ 315 [M-C_5_H_10_O_4_]^+^	1.37	1.32	4.83
2	Petunidin 3-glucoside (Pet 3-glc)	C_22_H_23_O_12_^+^	478	479 [M + H]^+^	317 [M-C_6_H_11_O_5_]^+^ 302 [M-C_6_H_11_O_4_]^+^	1.07	1.01	7.15
3	Peonidin 3-glucoside (Peo 3-glc)	C_22_H_23_O_11_^+^	462	463 [M + H]^+^	301 [M-C_6_H_11_O_5_]^+^ 201 [C_9_H_5_O_4_]^+^	1.15	1.03	5.70
4	Malvidin 3-glucoside (Mv 3-glc)	C_23_H_25_O_12_^+^	492	493 [M + H]^+^	331 [M-C_6_H_11_O_5_]^+^ 315 [M-C_5_H_10_O_4_]^+^	1.22	1.08	6.73
5	Cyanidin 3-(acetylglucoside) (Cy 3-acglc)	C_23_H_23_O_12_^+^	490	491 [M + H]^+^	287 [M-C_8_H_13_O_6_]^+^ 163 [M-C_17_H_13_O_7_]^+^	1.20	1.21	6.78
6	Malvidin 3-(6″acetyl) glucoside (Mv 3-(6-acglc))	C_25_H_27_O_13_^+^	534	535 [M + H]^+^	331 [M-C_8_H_13_O_6_]^+^ 315 [M-C_8_H_13_O_7_]^+^	1.12	1.03	8.47
7	Petunidin 3-(6″-cumaroyl)-glucoside (Pet 3-(cum)glc)	C_31_H_29_O_14_^+^	624	625 [M + H]^+^	317 [M-C_15_H_17_O_7_]^+^ 301 [M-C_15_H_18_O_8_]^+^	1.53	1.31	9.69
8	Malvidin 3-(6″-cumroyl)- Glucoside (Mv 3-(6-cum)glc)	C_32_H_31_O_14_^+^	638	639 [M + H]^+^	331 [M-C_15_H_17_O_7_]^+^ 447 [M-C_6_H_10_O_5_]^+^	1.71	1.35	7.32
9	Peonidin 3-(6″-cumaroyl)-glucoside (Peo 3-(6-cum)glc)	C_31_H_29_O_13_^+^	608	609 [M + H]^+^	301 [M-C_15_H_17_O_7_]^+^ 492 [M-C_9_H_8_O_4_]^+^	1.71	1.45	8.18

**Table 4:** Effect of coco-glucoside concentration on total phenolic content (TPC), total anthocyanin content (TAC), and antioxidant activity (DPPH, ABTS) of red grape pomace extracts (mean ± SD).

Extract	CG Concentration [%]	TPC [mg GAE/L]	TAC [mg Cyd-3-glu/L]	DPPH [mg TE/L]	ABTS [mg TE/L]
GPE_CG 1p	1	2631.7 ± 41.0	201.7 ± 8.2	3353.0 ± 49.8	3415.3 ± 57.0
GPE_CG 2p	2	2747.7 ± 37.4	226.8 ± 5.3	3598.6 ± 14.2	3708.6 ± 49.6
GPE_CG 4p	4	2780.1 ± 31.2	203.9 ± 5.1	3152.5 ± 50.1	3411.7 ± 42.3

**Table 6:** Model cosmetic formulation (shampoo).

	Ingredient (INCI Name)	[% m/m]	
		Sh_E_0p	Sh_E_10p
1	Sodium Lauryl Sulfate, Ammonium Lauryl Sulfate, Disodium 2-Sulfolaurate (Anionic surfactants)	9	
2	Coco-Glucoside (Non-ionic surfactant)	2	1.8
3	Cocamidopropyl Betaine (Amphoteric surfactants)	2	
4	Citric Acid (pH regulator)	to pH 5.5	
5	Benzyl Alcohol, Benzoic Acid, Dehydroacetic Acid, Tocopherol (Preservative)	0.5%	0.45%
6	Guar Hydroxypropyltrimonium Chloride (Conditioning polymer)	0.1	
7	Extract	0	10
	*Coco-Glucoside*	*0*	*0.2*
	*Preservative*	*0*	*0.05*
	*Aqua*	*0*	*9.76*
8	Aqua-to 100	to 100	
9	Sodium Chloride (Viscosity modifier)	2	

**Table 7:** Basic properties of designed cosmetics products.

Cosmetics Product	Stability	Viscosity mPa·s	Foaming Ability cm^3^	Foam Stability %	Irritating Potential mgN/100 mL
Sh_E_0p	stable	3392 ± 180	384 ± 25	84	230.7 ± 0.5
Sh_E_10p	stable	3392 ± 180	304 ± 20	95	221.4 ± 0.5

## Data Availability

Data is contained within the article.

## Abbreviations

The following abbreviations are used in this manuscript:

APGs	alkyl polyglucosides
CG	coco-glucoside
DG	decyl glucoside
